# The Neglect and the Value of Blistering

**Published:** 1879-09

**Authors:** 


The Neglect and the Value of Blistering.—Dr. H. S. Anderson, in his Harveian Discourse, published in the Edingburg *Medical Journal,* says:

A remedy which I fear is somewhat unduly neglected now-a-days is counter-irritation by means of blistering; and I think I have observed in some young practioners an approach to something like terror when blistering is spoken of as a remedy that may frequently be used. Certainly, as regards children's diseases, there is more of this fear than there should be. It has frequently, for example, been my experience to see children, in consultation with a younger practioner, when blistering in acute head affection had never been dreamed of. In mostly all acute inflammatory affections of the brain, tubercular or not, in children, I am strongly of opinion, that, after shaving the head, the application of blistering fluid has a most rapid and satisfactory effect. Inflammatory attacks also, of the peritoneum and chest in children, are often controlled by blistering, although the size of the vesicatory and the length of time applied must be carefully considered. And in the rheumatic affections of the joints, in adults, repeated blistering has often the happiest results. For many chronic conditions also, counter-irritation has always held a high place in my list of remedies. In chronic tubercular affections of both chest and abdomen, I think occasional and repeated blistering is frequently beneficial, and also in chronic and obscure head and other affections of the nervous system. For example, a blister over the roots of the nerves, in herpse zoster, often relieves the neuralgic pain so generally present, and often so difficult to get rid of. In diptheritic paralysis, also, blistering the nape of the neck, and even down the spine, often expedites cure in a wonderful way. In the uterine or ovarian pain so often complained of in the left side, there is no better remedy sometimes than a succession of fly-blisters, and the tenderness of the spinal irritation is very frequently relieved, if not got rid of, by the same means. In chronic effusion the use of the blisters is still more fully cknowledged, and does not therefore, call for special attention.—*Canada Med. Journal.*

